# Use of Smartphones for Early Detection of Melanoma: Systematic Review

**DOI:** 10.2196/jmir.9392

**Published:** 2018-04-13

**Authors:** Cédric Rat, Sandrine Hild, Julie Rault Sérandour, Aurélie Gaultier, Gaelle Quereux, Brigitte Dreno, Jean-Michel Nguyen

**Affiliations:** ^1^ Department of General Practice Faculty of Medicine University of Nantes Nantes France; ^2^ Unit 1232 – Team 2 Centre de Recherche en Cancérologie French National Institute of Health and Medical Research Nantes France; ^3^ Department of Epidemiology and Biostatistics Nantes University Hospital CHU Nantes Nantes France; ^4^ Oncodermatology Department Nantes University Hospital CHU Nantes Nantes France

**Keywords:** smartphone, melanoma, screening, teledermatology, telemedicine, mobile app

## Abstract

**Background:**

The early diagnosis of melanoma is associated with decreased mortality. The smartphone, with its apps and the possibility of sending photographs to a dermatologist, could improve the early diagnosis of melanoma.

**Objective:**

The aim of our review was to report the evidence on (1) the diagnostic performance of automated smartphone apps and store-and-forward teledermatology via a smartphone in the early detection of melanoma, (2) the impact on the patient’s medical-care course, and (3) the feasibility criteria (focusing on the modalities of picture taking, transfer of data, and time to get a reply).

**Methods:**

We conducted a systematic search of PubMed for the period from January 1, 2007 (launch of the first smartphone) to November 1, 2017.

**Results:**

The results of the 25 studies included 13 concentrated on store-and-forward teledermatology, and 12 analyzed automated smartphone apps. Store-and-forward teledermatology opens several new perspectives, such as it accelerates the care course (less than 10 days vs 80 days), and the related procedures were assessed in primary care populations. However, the concordance between the conclusion of a teledermatologist and the conclusion of a dermatologist who conducts a face-to-face examination depended on the study (the kappa coefficient range was .20 to .84, median κ=.60). The use of a dermoscope may improve the concordance (the kappa coefficient range was .29 to .87, median κ=.74). Regarding automated smartphone apps, the major concerns are the lack of assessment in clinical practice conditions, the lack of assessment in primary care populations, and their low sensitivity, ranging from 7% to 87% (median 69%). In this literature review, up to 20% of the photographs transmitted were of insufficient quality. The modalities of picture taking and encryption of the data were only partially reported.

**Conclusions:**

The use of store-and-forward teledermatology could improve access to a dermatology consultation by optimizing the care course. Our review confirmed the absence of evidence of the safety and efficacy of automated smartphone medical apps. Further research is required to determine quality criteria, as there was major variability among the studies.

## Introduction

### Background

The incidence of melanoma has increased in all Western countries over the last 30 years and has increased 3 to 5 times depending on the country [1,2], presently affecting 13.8 people in North America, 14.6 in northern Europe and 35.1 out of the 100,000 people in Australia [1,2]. Melanoma 5-year survival depends on the stage at the time of diagnosis, decreasing by 84% at a localized stage to 13% at the metastatic stage [2]. It is therefore essential that clinicians and policy makers concentrate their efforts to ensure early detection of the disease [3]. Numerous factors associated with a delayed diagnosis are patient-related [4-12]. Other factors are related to the opportunity to consult a dermatologist rather than a general practitioner [4,13]. However, various authors have reported difficulties in obtaining an appointment with a dermatologist [12,14-17].

Many countries have tested the use of telemedicine in dermatology as a way to increase access to health care services when distance is a critical factor [18-26]. Telemedicine in dermatology can be based either on videoconferences or on store-and-forward teledermatology procedures. Videoconferences, which allow a patient and a dermatologist to be connected for a consultation, are time-consuming for the dermatologist and may require an expensive setup [20,27]. Store-and-forward teledermatology procedures are based on sending information and photographs to a dermatologist for a deferred medical opinion [20].

Although smartphones have now revolutionized the daily life of physicians in all Western countries [19,24,28,29], one issue is to determine whether smartphone use has been assessed for store-and-forward teledermatology procedures. As various apps exist that provide scores, decision aids, and management advice, an alternative both to videoconferences and to store-and-forward teledermatology might be automated smartphone apps with no need of a dermatologist opinion. An issue to be addressed is whether such apps can help in the early diagnosis of melanoma.

### Objective

We initiated a review focusing on the use of a smartphone in sustaining melanoma early detection (either store-and-forward teledermatology or automated apps). The aim was to report evidence on (1) the diagnostic performance of the procedures, (2) the impact on the patient’s medical-care course and delays before the dermatological consultation, and (3) the limitations of either store-and-forward teledermatology or automated apps.

## Methods

This review was conducted according to the key steps required for systematic reviews [30]. Considering that evidence might be sparse, the literature review was based on a broad scope and was not restricted to randomized controlled trials.

### Study Identification and Selection

We conducted a systematic search of PubMed for the period from January 1, 2007 (launch of the first smartphone) to November 1, 2017. The keywords were as follows: [smartphone OR cell phone OR remote OR telemedicine] AND [dermatology OR skin disease OR melanoma OR skin neoplasm OR skin abnormalities]. We also searched the reference lists of reviews and studies identified during the initial search by hand. Abstracts and full texts were reviewed independently by 2 reviewers (SH and JR) for inclusion. Any disagreements on inclusion or exclusion were resolved by consensus, and a third reviewer (CR) was consulted to resolve any remaining disagreements.

### Inclusion and Exclusion Criteria

In this manuscript, the term “smartphones” refers to mobile phones that have an internet data communication system and a digital camera (compared to mobile phones that would not have these two specific devices). The inclusion criteria for the studies included in this review were as follows: (1) photographs concerning pigmented suspicious lesions, (2) photographs taken or analyzed using a smartphone, (3) patients older than 18 years, (4) studies written in French or English, and (5) abstracts available.

The exclusion criteria were (1) no use of a smartphone, (2) the research area was not related to melanoma early detection, (3) dermatology teleconsultation in the form of a videoconference, (4) study based on histology, (5) studies consisting of assessing patients or caregivers’ preferences through interviews or surveys, and (6) an editorial or a letter to the editor.

Considering that evidence might be sparse, the literature review was based on a broad scope and the inclusion criteria were not restricted to a “Patients Intervention Comparison Outcome” presentation [30].

### Data Extraction and Synthesis

Studies were critically appraised by 2 reviewers (SH and JR), and discrepancies were resolved by consensus. The studies were first classified by the type of procedure assessed (app or store-and-forward teledermatology). Then the following data were extracted, such as design, population of the sample, whether the study had been conducted in the context of primary care, whether it was a descriptive or comparative study, and main outcome measures (Tables 1 and 2).

The main outcomes in studies focusing on store-and-forward teledermatology were either (1) the diagnostic concordance between the teledermatology procedure and the reference (the kappa coefficient of concordance is a measure of agreement between 2 raters, based on the following formula [(observed probability–expected probability)/(1–expected probability)]) or (2) the impact on the patient’s medical-care course and delays before dermatological consultation. The proportion of uninterpretable photographs was also reported.

The main outcomes in studies focusing on apps were sensitivity (defined as the number of true positive assessments/number of all positive assessments), specificity (defined as the number of true negative assessments/number of all negative assessments), and accuracy (defined as the number of correct assessments/number of all assessments). The proportion of uninterpretable photographs was also reported. When necessary, the authors were contacted to obtain information not reported in the studies.

### Analysis of Bias

We assessed the risk of bias using the quality assessment of diagnostic accuracy 2nd edition (QUADAS-2) [55]. The reporting of risk of bias focused on patient selection, index test, reference standard, and flow and timing. For each item, signaling questions helped to estimate whether the risk of bias was low or high. Unclear was used if no information was available. The applicability of the study intervention was also assessed using QUADAS-2, focusing on patient selection, index test, and reference standard.

**Table 1:** Studies based on store-and-forward teledermatology procedures (design, patients, comparison, and outcome). N/A: not applicable. RCT: randomized controlled trial.

Authors		Design	Patients, n (%)	Population of sample	Comparison	Main outcomes
**Store-and-forward teledermatology without teledermoscopy**						
	Boyce et al, 2011 [31]	Prospective study	N/A	Patients at an elevated risk of melanoma	Face to face	Concordance
	Lamel et al, 2011 [32]	Prospective study	1 (0.7)	Patients from a melanoma screening campaign	Face to face	Concordance
	Janda et al, 2014 [33]	RCT	1 (1)	Patients at an elevated risk of melanoma	Face to face	Concordance
**Store-and-forward teledermatology that included teledermoscopy**						
	Ford et al, 2015 [34]	Quasi-experiment	22 (11.3)	Patients recruited in primary care centers	Face to face	Secondary care referral
	Börve et al, 2013 [29]	Prospective study	12 (17)	Patients referred for an excision	Face to face^a^	Concordance
	Börve et al, 2015 [35]	Quasi-experiment	55 (3.52)	Patients recruited in primary care centers	Teledermatology versus paper referral	Delays
	Hue et al, 2016 [36]	Descriptive study	1 (0.3)	Patients from a melanoma screening campaign	No comparison	Feasibility and delays
	Janda et al, 2013 [37]	Descriptive study	N/A	Patients at an elevated risk of melanoma	No comparison	Feasibility
	Kroemer at al, 2011 [38]	Prospective study	6 (5)	Patients referred to the dermatologist	Face to face^a^	Concordance
	Manahan et al, 2015 [39]	RCT	0 (0.0)	Patients with a dermatological follow-up	Face to face	Concordance
	Markun et al, 2017 [40]	Prospective study	1 (0.05)	Patients from a melanoma screening campaign	Face to face^a^	Concordance
	Massone et al, 2007 [41]	Prospective study	2 (11)	Patients with a dermatological follow-up	Face to face	Concordance
	Wu et al, 2015 [42]	Prospective study	N/A	Patients with a dermatological follow-up	Face to face	Concordance

^a^For these studies, suspicious lesions were referred for excision and histopathology results were analyzed as a secondary outcome in the study.

**Table 2:** Studies based on automated smartphone apps (design, photographs and patients, comparison, and outcomes). N/A: not applicable.

Authors		Design	n (%)^a^	Patients, N (characteristics)	Comparison	Main outcomes
**Photographs issued from a database**						
	Do et al, 2014 [43]	Case-control study	29 (36)	N/A	Histopathology	Accuracy^b^
	Doukas et al, 2012 [44]	Case-control study	800 (26.67)	N/A	Clinical evaluation	Accuracy^b^
	Ferrero et al, 2013 [45]	Descriptive study	93 (100)	N/A	Histopathology	Sensitivity, Specificity
	Ramlakhan et al, 2011 [46]	Case-control study	46 (55)	N/A	Unclear	Sensitivity, Specificity
	Wadhawan et al, 2011a [47]	Case-control study	388 (29.85)	N/A	Histopathology	Sensitivity, Specificity
	Wadhawan et al, 2011b [48]	Case-control study	110 (31.7)	N/A	Histopathology	Sensitivity, Specificity
	Wolf et al, 2013 [49]	Case-control study	60 (31.9)	N/A	Histopathology	Sensitivity, Specificity
**Photographs taken of patients in real condition**						
	Dorairaj et al, 2017 [50]	Prospective study	9 (28)	N/A (referred for an excision)	Teledermatologist^c^	Sensitivity, Specificity
	Maier et al, 2015 [51]	Prospective study	26 (18.1)	N/A (with a dermatological follow-up)	Face to face^c^	Sensitivity, Specificity
	Ngoo et al, 2017 [52]	Prospective study	1 (2)	30 (with a dermatological follow-up)	Teledermatologist	Sensitivity, Specificity
	Robson et al, 2012 [53]	Prospective study	2 (6)	31 (referred to the dermatologist)	Face to face^c^	Sensitivity, Specificity
	Thissen et al, 2017 [54]	Prospective study	6 (1.8)	256 (referred to the dermatologist)	Face to face^c^	Sensitivity, Specificity

^a^Included photographs, proportion with melanoma.

^b^Accuracy=(True Negatives+True Positives)/(True Negatives+True Positives+False Negatives+False Positive).

^c^For these studies, suspicious lesions were referred for excision and histopathology results were analyzed as a secondary outcome in the study.

## Results 

### Overview

**Figure 1:**
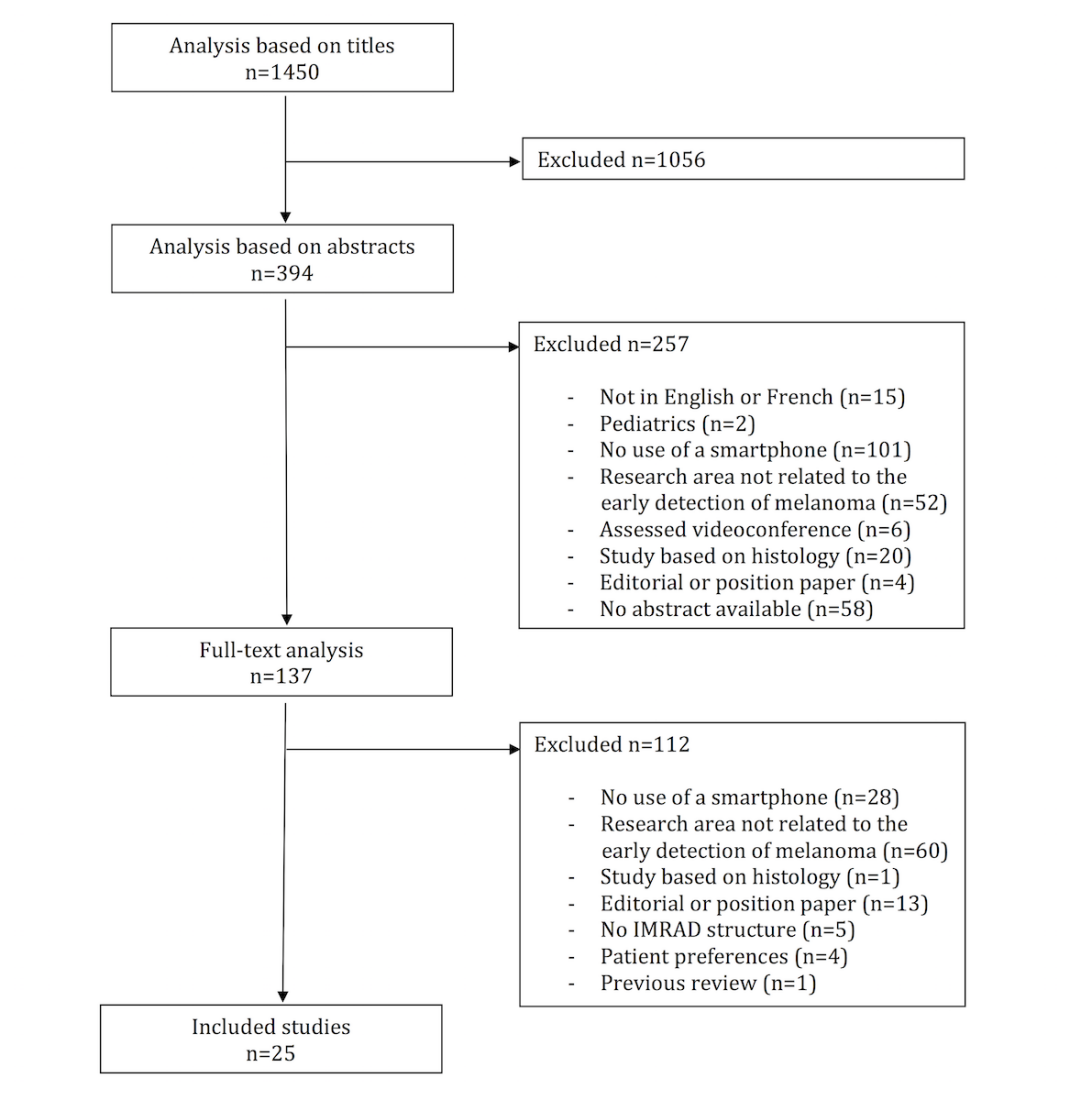
Flowchart of studies identified in this systematic review focusing on the use of smartphones in the early detection of melanoma. IMRAD: Introduction, Methods, Results, And Discussion.

In total, 1450 titles and abstracts were screened for eligibility, utilizing the inclusion and exclusion criteria. A previous review was identified [2] and the related studies from the references list were included in this review. A total of 25 studies [29,31-54,56] were included in the review (Figure 1). Of these, 15 studies had been published as original papers [29,31,32,34-36,38-42,49,51,52,54], 5 were conference papers [43,44,46-48] and 5 were research letters [33,37,45,50,53]. In these, 12 studies had been conducted in European countries, that is, Great Britain [34,53], Austria [38,41], Sweden [29,35], Ireland [50], Germany [51], Switzerland [40], Greece [44], the Netherlands [54], and France [36]; 7 in United States of America [32,42,45-49]; 5 in Australia [31,33,37,39,52]; and one in Singapore [43].

### Store and Forward Teledermatology

A total of 13 studies assessed store-and-forward teledermatology [29,31-42] (Table 1). There were 12 studies that specified the smartphone model used, that is, 9 tested iPhones [29,33-37,39,40,42] and 3 tested other brands of telephones [32,38,41].

The study population were patients recruited in primary care in 9 studies, that is, either in the context of a screening campaign [32,36,40] or during targeted screening focusing on patients at an elevated risk of melanoma [31,33,37] or during opportunistic screening conducted in general practice [34,35,38]. For the other 4 studies, the patients had already consulted a dermatologist [29,39,41,42]. The prevalence of melanomas in the related populations varied greatly, ranging from 0% [39] to 17.3% [29].

Ten studies compared the conclusion of the teledermatologist with the conclusion of a dermatologist who conducted a face-to-face examination (Table 1). There were 2 studies that focused on feasibility without providing any comparison [36,37]. From the 13 studies conducted, 10 studies used a mobile teledermoscope [29,34-42], whereas 3 studies only transferred the pictures taken without the teledermoscope [31-33].

There were 7 studies that provided information on diagnostic concordance for store-and-forward teledermatology based solely on clinical photographs. The diagnostic concordance between the conclusions of the teledermatologist and the dermatologist (face-to-face) ranged from 62% [32] to 89% [41]. This concordance was analyzed further using the kappa coefficient [29,31,32,38-40,42], which ranged from .20 [40] to .84 [38]. Börve reported 58% concordance between the conclusions of 2 independent teledermatologists [29]. Focusing on whether the patients could take pictures of their lesions themselves, Boyce et al reported 69% concordance between the conclusion of a dermatologist (face-to-face) and the conclusion of a teledermatologist (κ=.23) [31].

For teledermatology based on pictures taken with a teledermoscope, the diagnostic concordance between the teledermatologist’s conclusions and the conclusion of a dermatologist (face-to-face) ranged from 51% [29] to 97% [42]. The kappa coefficient varied from .29 [40] to .87 [42]. Massone reported 94% concordance between the conclusions of 2 independent teledermatologists who analyzed photographs taken using a teledermoscope [41]. Focusing on whether the patients could take pictures of their lesions themselves, Manahan and Wu reported concordance ranging from 90% [39] to 97% [42] when the patient used a teledermoscope.

There were 4 studies that reported an acceleration in the management of patients when malignancy was suspected [29,34,36,40]. Börve reported reduced delays in obtaining an appointment (delays shorter than 2 days compared with delays longer than 80 days), a reduced delay for surgical management (36 days vs 85 days), and a lower Breslow index at the time of the diagnosis [29]. Hue reported that patients with a highly suspicious lesion were asked to return within less than 10 days [36]. There were 3 studies [35,36,40] that reported a decrease (40%, 53%, and 74%, respectively) in the proportion of patients referred to a dermatologist, whereas Ford reported a slight increase in referrals (an increase of 2.11 per 1000 patients) [34].

The proportion of uninterpretable images due to their poor quality was, on average, less than 20% [29,40,42]. Only Massone et al (2007) reported a higher percentage of poor-quality images of 70% [41]. However, only a minority of authors provided information on the modalities for picture taking. A total of 4 studies specified the size of the pictures from 1024×766 to 2592×1224 pixels. Following contact with the authors, the following information on the modalities were collected, that is, the number of pictures could vary from 1 to 12 pictures per lesion [33,39], pictures were taken at a distance of 10 to 30 cm from the skin [29,40], authors reported taking one close-up picture and another of the surrounding area [36,40,41], and specified the lighting conditions (“strong light” [39], “day light” [41], “maximal light” [29]), neutral background [29,40], use of the zoom [41] or macro mode [40,41], and use of the autofocus [29,38,41]). There were 4 authors who reported the time required to take the picture that is, ranging from a few seconds to less than 4 min [29,34-36]. The photographer was a professional in most cases, that is, either a dermatologist [29,38,41], a general practitioner [35], or another professional [32,40]; however, 6 studies reported that the picture was taken by the patient or a family member [31,33,37,39,42]. The notion of encrypting data was not approached systematically. Following individual contact, 8 authors reported encryption of the data, either through the app or through anonymity [29,31,32,34-36,39,40,42]. A total of 8 studies used email for transferring the data [29,31,33-35,37,39,42], and 7 used an encrypted platform [29,32,34-36,40,42].

Analysis of bias is provided in Table 3. The risk of bias related to patient selection was high, as patients who participated were either volunteers or chosen by doctors in consultation, but no study was based on a random sample. In 2 studies, the same dermatologist participated in both, the store-and-forward teledermatology procedure and the face-to-face clinical evaluation, with insufficient washout, so that the risk of bias related to the test index was high. The final analysis of the studies did not include all the recruited patients so that the risk of bias of flow and timing was high. Applicability was good.

### Automated Smartphone Apps

A total of 12 studies assessed the performance of automated smartphone apps [43-54] (Table 2), that is, 1 study compared the conclusions of an automated app with the conclusions of a dermatologist who conducted a face-to-face examination [51], 1 study compared the conclusions of an automated app with the conclusions of a teledermatologist [52], 7 studies analyzed the images of an already classified data bank [43-49], and the 3 last ones compared the conclusion of an automated app, both with the pathological report (after excision of the lesion) and with the conclusion of teledermatologists [50,53,54].

Among the 5 studies based on taking a photograph [51-54], 4 tested iPhones [51-54], 1 tested other brands of telephones [52], and the brand of the telephone was not specified in the last study [50]. The photographer was a dermatologist [53,54] or another professional [50-52].

Participants who were recruited in studies assessing automated smartphone apps were highly selected. Among the 8 studies based on photographs issued from a database, the proportion of melanoma ranged from 26.7% [44] to 100% [45]. Among the 5 studies based on taking a photograph, 2 studies included patients from a primary-care setting recruited during an opportunistic screening campaign conducted in general practice [53,54]. For the other 3 studies that included patients, the patients had already consulted a dermatologist [50-52]. The prevalence of melanomas in the related populations ranged from 1.8% [52,54] to 28.1% [50].

The performance of the automated smartphone apps were assessed by referring either to their capacity to classify the lesions at risk [43,45,47,49-54], or to their diagnostic capacity [44,46,48] (Table 2). The references used could be either the histology results in 5 studies [43,45,47-49], the teledermatologist’s conclusion in 2 studies [50,52], or the dermatologist (face-to-face) conclusion in 3 studies [51,53,54]. For 2 studies, the authors referred to histology-based diagnosis without describing how they obtained the histopathological conclusion [44,46].

A total of 5 studies assessed the apps used in real conditions [50-54]. In 4 studies, the images came from medical [43,45,49] or commercial [47,48] data banks, whereas no details on the photograph data banks were provided for 2 studies [44,46]. The sensitivity ranged from 7% to 87% [48,49], and the specificity ranged from 9% to 100% [50,52]. Only 2 studies described the area under the curve [44,48], providing a better comparison of results. One study reported a kappa coefficient of concordance between the opinion of the app and that of a dermatologist [52].

None of the studies that assessed automated smartphone apps reported an impact on the patient’s medical-care course.

Analysis provided by automated smartphone apps were made difficult by ulcerated, blood stained, speckled or tanned areas, the presence of hair, or several lesions on the same photograph. There were 5 studies that had a proportion ranging from 11% to 30% of the lesions that could not be analyzed because of technical problems other than the problems related to the quality of the initial photograph [45,49-51,53]. There were 4 studies based on apps which reported that the time required to analyze the pictures was less than 10 seconds [44,46-48]. The notion of encrypting data was not approached systematically. Following individual contact, 2 authors [52-54] reported encryption of the data, either through the app or through anonymity.

The analysis of bias is provided in Table 4. For studies based on photographs from databank, references were unknown and the risks of bias for patient selection were high. Applicability related to the patient selection was highly concerning. Automated apps had no information on the diagnosis so that the risk of bias related to the test index was low. In 3 studies, pictures were modified before intervention so that the applicability related to the index test was not good. All the photographs were not analyzed so that the risk of bias related to flow and timing was high.

**Table 3:** Studies based on store-and-forward teledermatology procedures. Risk of bias assessment according to quality assessment of diagnostic accuracy 2nd edition (QUADAS-2).

Authors		Risk of bias				Applicability concerns		
		Patient selection	Index test	Reference standard	Flow and timing	Patient selection	Index test	Reference standard
**Store-and-forward teledermatology without teledermoscopy**								
	Boyce et al, 2011 [31]	High	Low	Low	High	Low	Low	Low
	Lamel et al, 2011 [32]	High	Low	Low	Low	Low	Low	Low
	Janda et al, 2014 [33]	High	Low	High	Low	Low	Low	Low
**Store-and-forward teledermatology that included teledermoscopy**								
	Ford et al, 2015 [34]	Low	Low	Low	High	Low	Low	Low
	Börve et al, 2013 [29]	High	Low	Low	Low	High	Low	Low
	Börve et al, 2015 [35]	High	Low	Low	High	Low	Low	Low
	Hue et al, 2016 [36]	High	Low	Low	High	Low	Low	Low
	Janda et al, 2013 [37]	High	Low	Low	High	Low	Low	Low
	Kroemer at al, 2011 [38]	High	High	Low	High	Low	Low	Low
	Manahan et al, 2015 [39]	High	Low	Low	High	Low	Low	Low
	Markun et al, 2017 [40]	High	High	Low	High	Low	Low	Low
	Massone et al, 2007 [41]	High	Low	Low	Low	Low	Low	Low
	Wu et al, 2015 [42]	High	Low	Low	High	Low	High	Low

**Table 4:** Studies based on automated smartphone apps. Risk of bias assessment according to quality assessment of diagnostic accuracy 2nd edition (QUADAS-2).

Authors		Risk of bias				Applicability concerns		
		Patient selection	Index test	Reference standard	Flow and timing	Patient selection	Index test	Reference standard
**Photographs issued from a database**								
	Doukas et al, 2012 [44]	High	Low	Unclear	Unclear	High	Low	Unclear
	Do et al, 2014 [43]	High	Low	Low	Unclear	High	High	Low
	Ferrero et al, 2013 [45]	High	Low	Low	High	High	Low	Low
	Ramlakhan et al, 2011 [46]	High	Low	Unclear	High	High	Low	Unclear
	Wadhawan et al, 2011a [47]	High	Low	Low	High	High	High	Low
	Wadhawan et al, 2011b [48]	High	Low	Low	High	High	High	Low
	Wolf et al, 2013 [49]	High	Low	Low	High	High	High	Low
**Photographs taken of patients in real condition**								
	Dorairaj et al, 2017 [50]	High	Low	Low	High	High	Low	Low
	Maier et al, 2015 [51]	High	Low	Low	High	High	Low	Low
	Ngoo et al, 2017 [52]	High	Low	Low	High	Low	Low	Low
	Robson et al, 2012 [53]	High	Low	Unclear	High	Low	Low	Low
	Thissen et al, 2017 [54]	High	Low	Unclear	Low	Low	Low	Low

## Discussion

Store-and-forward teledermatology opens several perspectives, that is, it accelerates the care course, and various studies were performed in primary care populations. However, the concordance between the conclusion of a teledermatologist and the conclusion of a dermatologist who conducts a face-to-face examination depended on the study (the kappa coefficient range was .20 to .84, median κ=.60). The use of a dermoscope may improve the concordance (the kappa coefficient range was .29 to .87, median κ=.74). Regarding automated smartphone apps, the major concerns are their low sensitivity, the lack of assessment in clinical practice conditions, and the lack of assessment in primary care populations.

In a study recently published in *Nature*, Esteva et al reported that an artificial neuronal network had a better capacity to recognize melanomas than a dermatologist [56]. However, the automated apps available on a smartphone in 2017 do not provide such expertise. Our review shows that the existing automated smartphone apps are unreliable. Some apps have a low diagnostic sensitivity that may induce false negatives and erroneously reassure patients who may then not consult a specialist [50]. Certificates do not guarantee good diagnostic performance [54]. Greater control by administrative authorities is necessary [45,57,58].

This literature review suggests that teledermatology decreases the delays in the management of melanoma lesions [34-36,40,59], reduces the referrals to a dermatologist by avoiding unnecessary consultations [35,36,40] and limits the number of patients lost from follow-up [36,60]. Moreno-Ramirez et al confirmed this point during an experimental teledermatology program without a smartphone [61]. Other authors have not been as optimistic and suggested that the many false negatives and easy access to a dermatologist’s opinion may increase the number of secondary consultations [34].

Large variations in the kappa concordance coefficient might be related to the range of melanoma prevalence (depending on the recruited population). On the one hand, this result should lead to multiple studies in the general population to assess the performance of the procedures in a primary care setting. On the other hand, this review emphasizes that the publication of data related to up-to-date technologies is a challenge in teledermatology [20,62]; it is notable that up to 70% of the photographs were of poor quality in a study performed in 2007 [41], whereas all photographs were interpretable in 2016 [54].

The importance of training the person taking the photograph has been underlined [33,35-37,40], especially when a dermoscope is used [63]. Today, it is surprising to note that no standards for taking photographs exist [62,64]. Our review identified a few characteristics related to the quality of the photographs, such as several views of the same lesion, close-up and distant pictures, use of the autofocus and macro mode, a neutral background, and good lighting. The homogenization of practices based on these quality criteria is required to obtain better study reproducibility.

This review recalls that to give an opinion solely based on photographs, whatever their quality, is a challenge for dermatologists [29,41,63]. For example, the absence of palpation is one of the limitations of teledermatology. Thissens’s study noted the need to obtain supplementary clinical information [54].

General practitioners and patients are likely to omit suspect lesions [31,33,39]. This limitation, which has been described in general practice [13,65], exists in teledermatology, that is, general practitioners could miss up to 30% of melanomas [66]. Another difficulty is the omission of specific areas that are either difficult to access, such as hair, wounds, or the ear [51,54], or those considered sensitive (genitalia) [67,68].

Store-and-forward teledermatology is well-accepted by patients and caregivers [59,69,70]. Patients report that one limitation of the apps is the difficulty of not having any human contact [20]. For both procedures, a limitation is the loss of the face-to-face patient-physician relationship, which may be critical since a melanoma is diagnosed early, at a severe stage, or in the aging population [59,69-71]. Positive perspectives may improve compliance to referrals and possibilities for the patient to participate actively in his or her health [24].

Confidentiality, security, and traceability of data exchange are the major ethical and legal stakes [20,63,72]. Although the abusive use of clinical photographs has become an increasing preoccupation of health fund organizations [73], only 30% of patients worry about the future of their photographs [74], and our review reported that only slightly more than half of the authors encrypted their data. Although 60% of specialists continue to store photographs of their patients in their personal mobile phones [72,74-77], one perspective could be to develop recourse to encrypt the medical-image libraries [62].

This review is original because of its specific focus on (1) melanoma early detection (mortality issues are not comparable for other dermatological pathologies) [20,62,78,79], (2) a primary care perspective (the sensitivity and specificity of a test depend on the prevalence of the disease), and (3) the use of a smartphone, that is, a tool implemented worldwide at low cost (not comparable to other expensive videoconference procedures) without limiting the study selection to apps [20,78-80]. However, this study had several weaknesses. First, this review had a large scope because we hypothesized that evidence might be sparse—the heterogeneity in study designs, the populations, the end points, the references used, and the presentations of the results made data comparison difficult. Second, the review was only based on MEDLINE. Third, the selection bias was high, and the prevalence of melanoma, which ranged from 0% to 100%, depended on the population studied. Fourth, the material used differed from one study to the next and from year to year, hence introducing a bias in evaluation.

Our review confirmed the absence of evidence of the safety and efficacy of smartphone medical apps. In contrast, our review found evidence that store-and-forward teledermatology using smartphones may affect patients’ care courses, delays in obtaining a dermatologist consultation, and patients’ referral to secondary care. Further research is required to determine the quality criteria, as there was major variability among the studies.

## Abbreviations

QUADAS-2: quality assessment of diagnostic accuracy 2nd edition
